# Treatment Induced Cytotoxic T-Cell Modulation in Multiple Myeloma Patients

**DOI:** 10.3389/fonc.2021.682658

**Published:** 2021-06-15

**Authors:** Gregorio Barilà, Laura Pavan, Susanna Vedovato, Tamara Berno, Mariella Lo Schirico, Massimiliano Arangio Febbo, Antonella Teramo, Giulia Calabretto, Cristina Vicenzetto, Vanessa Rebecca Gasparini, Anna Fregnani, Sabrina Manni, Valentina Trimarco, Samuela Carraro, Monica Facco, Francesco Piazza, Gianpietro Semenzato, Renato Zambello

**Affiliations:** Department of Medicine (DIMED), Hematology and Clinical Immunology Section, Padua University School of Medicine, Padova, Italy

**Keywords:** multiple myeloma, immunophenotyping, treatment, lenalidomide, ASCT-autologous stem cell transplantation, cytotoxic response

## Abstract

The biology of plasma cell dyscrasias (PCD) involves both genetic and immune-related factors. Since genetic lesions are necessary but not sufficient for Multiple Myeloma (MM) evolution, several authors hypothesized that immune dysfunction involving both B and T cell counterparts plays a key role in the pathogenesis of the disease. The aim of this study is to evaluate the impact of cornerstone treatments for Multiple Myeloma into immune system shaping. A large series of 976 bone marrow samples from 735 patients affected by PCD was studied by flow analysis to identify discrete immune subsets. Treated MM samples displayed a reduction of CD4+ cells (p<0.0001) and an increase of CD8+ (p<0.0001), CD8+/DR+ (p<0.0001) and CD3+/CD57+ (p<0.0001) cells. Although these findings were to some extent demonstrated also following bortezomib treatment, a more pronounced cytotoxic polarization was shown after exposure to autologous stem cell transplantation (ASCT) and Lenalidomide (Len) treatment. As a matter of fact, samples of patients who received ASCT (n=110) and Len (n=118) were characterized, towards untreated patients (n=138 and n=130, respectively), by higher levels of CD8+ (p<0.0001 and p<0.0001, respectively), CD8+/DR+ (p=0.0252 and p=0.0001, respectively) and CD3+/CD57+ cells (p<0.0001 and p=0.0006, respectively) and lower levels of CD4+ lymphocytes (p<0.0001 and p=0.0005, respectively). We demonstrated that active MM patients are characterized by a relevant T cell modulation and that most of these changes are therapy-related. Current Myeloma treatments, notably ASCT and Len treatments, polarize immune system towards a dominant cytotoxic response, likely contributing to the anti-Myeloma effect of these regimens.

## Introduction

The biology of plasma cell dyscrasias (PCD) involves both genetic and immune-related factors (1). The pathogenesis of Multiple Myeloma (MM) is a multi-step process in which primary events occurring in the plasma cell are responsible for immortalization and development of a pre-neoplastic condition defined as monoclonal gammopathy of undetermined significance (MGUS). The acquisition of secondary additional events sets the transition to an overt neoplastic and initially asymptomatic condition (smoldering Multiple Myeloma, SMM) and then to an active MM (AMM) requiring treatment (2–4). However, whole-exome sequencing studies of paired samples from MGUS, SMM and AMM patients demonstrated that most somatic mutations preceded the diagnosis of MM, suggesting that genetic lesions are necessary but not sufficient for the evolution from a pre-neoplastic condition to an overt neoplastic disease (5, 6). Consequently, several authors hypothesized that a permissive tumor microenvironment and more specifically an immune dysfunction also plays a key role in MM pathogenesis, suggesting that immune system impairment contributes to the progression of the disease (7, 8). Quantitative and functional alterations involving Natural Killer cells (NK cells), B and T lymphocytes are well known and described in previous studies (8–10).

As far as the B cell counterpart is concerned, immune-paresis is a well-known risk factor of progression from SMM to AMM (11) and secondary hypogammaglobulinemia contributes to infective events that represent a common clinical feature in the setting of symptomatic MM getting worse patients’ survival (12, 13). NK cells are functionally and phenotypically altered in PCD and progression of MGUS to MM is characterized by reduction of cytotoxic properties and acquisition of an “exhausted” phenotype (14, 15). Also the impairment of T cell compartments involving both CD4+ and CD8+ lymphocytes contributes to Myeloma pathogenesis with progressive reduction in cytotoxic properties and acquisition of an exhausted or anergic state (7, 8, 16, 17).

Despite different studies have been focused on the characteristic of immune cells of patients with PCD, most of them included limited small cohorts of selected patients enrolled in clinical trial (17–21). Moreover, in most studies only peripheral blood samples have been analyzed (19, 20, 22), thus ruling the evaluation out of the bone marrow microenvironment in which neoplastic plasma cells grow. To address these unsolved questions, 976 bone marrow samples of a large cohort of 735 patients affected by PCD were studied through the characterization of the immune subsets. The aim of this study is to evaluate the impact of cornerstone treatments for Multiple Myeloma, namely autologous stem cell transplantation (ASCT) and novel agents like bortezomib and lenalidomide, into immune system shaping.

## Materials and Methods

### Study Population

A large series of 976 bone marrow samples from a cohort 735 patients affected by PCD including MGUS, smoldering Multiple Myeloma and active Multiple Myeloma followed at the Hematology Unit of Padua University Hospital was studied. Bone marrow samples were collected from October 2012 to November 2019 at different time points according to clinical practice, to confirm complete response or in case of progressive disease. Samples of patients affected by other PCD, i.e. IgM MGUS, Waldenström Macroglobulinemia, light chain amyloidosis, light/heavy chain deposition disease and POEMS syndrome were excluded from the study.

This study was conducted according to the guidelines of the Declaration of Helsinki, and approved by the Institutional Review Board of Azienda Ospedaliera di Padova (2491P, PD-MM-REG1). All patients gave written informed consensus prior to inclusion in the study.

### Flow Cytometry Analysis

Flow cytometry analysis was performed on fresh bone marrow samples. The frequency of lymphocyte subsets was assessed by flow cytometry analysis using direct or indirect immunofluorescence assay combining 6 fluorescences. Briefly, cells were stained with the appropriate mAbs, scored using a FACS Canto analyzer (BD Biosciences, San Jose CA) and data processed by the BD FACS Diva software program (BD Biosciences). Bone marrow lymphocytes were stained with fluorochrome conjugated antibodies for CD4 (FITC, clone SK3), CD8 (PE, clone SK1), HLA-DR (PerCP, clone L243), CD5 (PeCy7, clone L17F12), CD19 (APC, clone SJ25C1), CD57 (FITC, clone HNK-1), TCRγδ (PE, clone 11F2), CD16 (PerCP-Cy5.5, clone 3G8), CD56 (PeCy7, clone NCAM 16.2), CD3 (APC, clone SK7) and CD45 (APC-Cy7, clone 2D1) (BD Biosciences). Based on these combinations, the following subsets of lymphocytes were identified: CD3+ T, CD4+ T cells, CD8+ T cells, CD8+/DR+ T cells, CD3+/CD57+ T cells and CD3+/Tγδ+ cells, CD19+ B lymphocytes and CD19+/CD5+ B cells, CD3-/CD16+/CD56+ NK cells along with the CD3-/CD16+/CD56+/CD57+ NK cell subset.

### Statistical Analysis

Data are expressed as mean plus or minus the standard deviation (SD), and statistical analysis was performed by t-test or by one-way Anova followed by Tukey’s multiple comparison test, when appropriated. All the analyses were performed using GraphPad Prism 6. A p-value <0.05 was accepted as significant.

## Results

### Distribution of Immune Subsets in Plasma Cells Dyscrasias

Clinical features of the cohort are reported in **Tables 1** and **2**. The 976 consecutive bone marrow samples of 746 patients’ samples collected from October 2012 to November 2019 were distributed as follows: 167 samples from 164 MGUS patients, 224 samples from 206 SMM patients and 585 samples from 376 AMM patients. Among the 585 AMM samples, 254 were from newly diagnosed patients (NDMM), while the remnant 331 belonged to treated patients (TMM); in detail n=192 (58.3%) received Autologous Stem Cell Transplantation (ASCT), n=300 (90.6%) bortezomib (Bort) treatment and n=118 (35.6%) lenalidomide (Len) treatment (**Table 2**). Bone marrow samples of treated patients were performed according to clinical practice, to confirm complete response or disease progression.

**Table 1:** Clinical features of the study cohort.

	MGUS (n = 167)	sMM (n = 224)	nMM (n = 254)	tMM (n = 331)
**Median Age (years)**	62 (24-93)	67 (35-85)	69 (39-90)	65 (38-87)
**Sex**				
** Female**	73/167 (43.7%)	96/224 (42.9%)	118/254 (46.5%)	156/331 (47.1%)
** Male**	94/167 (53.3%)	128/224 (57.1%)	136/254 (53.5%)	175/331 (52.9%)
**Isotype**				
** IgG**	112/167 (67.1%)	144/224 (64.3%)	152/254 (59.8%)	211/331 (63.7%)
** IgA**	36/167 (21.5%)	57/224 (24.4%)	49/254 (19.3%)	44/331 (13.3%)
** IgD**	0/167	0/224	4/254 (1.6%)	6/331 (1.8%)
** Light chain**	8/167 (4.8%)	13/224 (5.8%)	42/254 (16.5%)	47/331 (14.2%)
** Byphenotypical**	11/167 (6.6%)	10/224 (4.5%)	3/254 (1.2%)	6/331 (1.8%)
** Non-secretory**	0/167	0/224	4/254 (1.6%)	17/331 (5.1%)

MGUS, Monoclonal gammopathy of undetermined significance; sMM, smouldering Multiple Myeloma; nMM, newly diagnosed Multiple Myeloma; tMM, treated Multiple Myeloma.

**Table 2:** Clinical features of treated MM patients.

	Treated MM (n = 331)
**N° of previous treatment**	
** 1**	201/331 (60.7%)
** 2**	75/331 (22.7%)
** ≥3**	55/331 (16.6%)
**Type of previous treatment**	
** Bortezomib**	300/331 (90.6%)
Bortezomib alone*	77/331 (23.3%)
** Lenalidomide**	118/331 (35.6%)
** Pomalidomide**	14/331 (4.2%)
** Daratumumab**	10/331 (3.0%)
** Carfilzomib**	22/331 (6.6%)
** ASCT**	192/331 (58.3%)
* Within 3 months after ASCT*	83/331 (25.1%)

MM, Multiple Myeloma.

ASCT, autologous stem cell transplantation.

*With alkylating agents or dexamethasone.

**Figure 1:**
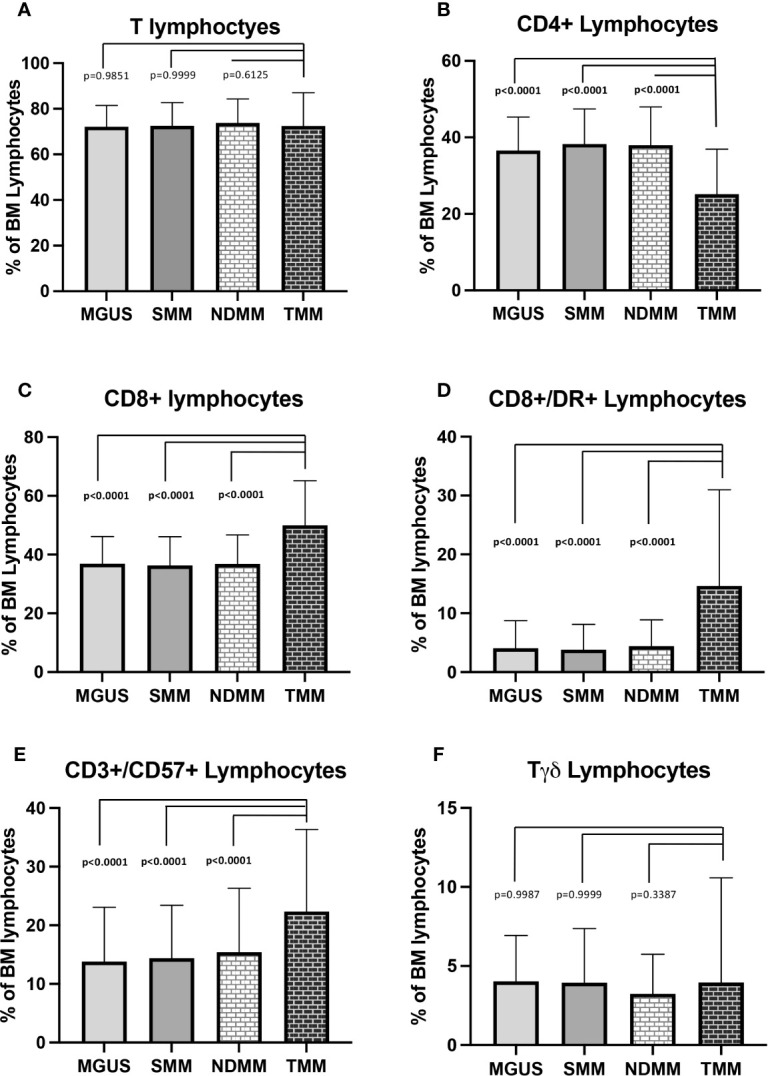
T cell subsets distribution in MGUS, sMM, nMM and tMM samples. **(A)** total T cells. **(B)** CD4+ Lymphocytes. **(C)** CD8+ Lymphocytes. **(D)** CD8+/DR+ lymphocytes. **(E)** CD3+/CD57+ Lymphocytes. **(F)** Tγδ lymphocytes. The comparisons between mean percentages were made by Anova followed by Tukey’s multiple comparison test. For all plots data are expressed as mean ± standard deviation. MGUS, monoclonal gammopathy of undetermined significance; SMM, smouldering Multiple Myeloma; NDMM, newly diagnosed Multiple Myeloma; TMM, treated Multiple Myeloma.

Considering total bone marrow lymphocytes, MGUS samples displayed reduced lymphocytes percentages towards NDMM samples (14.16 ± 5.7% vs 16.4 ± 8.7%, p=0.0285); no other significant differences were found in the remaining subsets. In TMM cases, no significant differences were found in total T cells percentages as compared to NDMM, SMM and MGUS patients (72.5 ± 14.6% vs 73.7 ± 10.5%, p=0.6125, 72.5 ± 14.6% vs 72.5 ± 10.2%, p=0.9999 and 72.5 ± 14.6% vs 72.1 ± 9.4%, p=0.9851, respectively, **Figure 1A**). Even though TMM samples did not show significant differences in total B lymphocytes levels with respect to NDMM, SMM and MGUS samples (10.5 ± 12.4% vs 9.5 ± 6.4%, p=0.5766, 10.5 ± 12.4% vs 10.7 ± 6.1%, p=0.9941 and 10.5 ± 12.4% vs 12.3 ± 7.5%, p=0.1721, **Supplementary Figure 1A**) a significant reduction in CD19+/CD5- B cells towards MGUS (7.4 ± 9.9% vs 10.4 ± 6.6%, p=0.0001, **Supplementary Figure 1B**) and a significant increase in CD19+/CD5+ B cells as compared to NDMM, SMM and MGUS was found (2.8 ± 4.1% vs 1.5 ± 2.6%, p<0.0001, 2.8 ± 4.1% vs 1.8 ± 2.5%, p=0.001 and 2.8 ± 4.1% vs 1.9 ± 2.5%, p=0.0136, respectively, **Supplementary Figure 1C**). Considering total NK cells and CD57+NK cells, no significant differences were found in TMM patients as compared to NDMM, SMM and MGUS patients (NK cells: 15.1 ± 10.3% vs 15.0 ± 8.4, p=0.9975, 15.1 ± 10.3% vs 14.2 ± 7.8%, p=0.6481 and 15.4 ± 9.5% vs 13.8 ± 7.2%, p=0.4369, respectively, **Supplementary Figure 1D**; CD57+ NK cells: 7.0 ± 7.3% vs 7.8 ± 5.8%, p=0.4696, 7.0 ± 7.3% vs 7.9 ± 6.2%, p=0.2939 and 7.0 ± 7.3% vs 6.7 ± 4.9%, p=0.9581, **Supplementary Figure 1E**).

Analysis of T cell subsets showed that TMM cases were characterized, with respect to NDMM, SMM and MGUS samples, by lower CD4+ lymphocytes percentages (25.2 ± 11.7 vs 38.0 ± 10.0, p<0.0001, 25.2 ± 11.7% vs 38.3 ± 9.1%, p<0.0001 and 25.2 ± 11.7% vs 36.6 ± 8.8% respectively, p<0.0001, **Figure 1B**) and higher CD8+ (49.9 ± 15.2% vs 36.8 ± 9.9%, p<0.0001, 49.9 ± 15.2% vs 36.3 ± 9.8%, p<0.0001 and 49.9 ± 15.2% vs 36.9 ± 9.3% respectively, p<0.0001, **Figure 1C**), CD8+/DR+ (14.7 ± 16.3% vs 4.4 ± 4.5%, p<0.0001, 14.7 ± 16.3% vs 3.8 ± 4.3%, p<0.0001 and 14.7 ± 16.3% vs 4.1 ± 4.7 respectively, p<0.0001, **Figure 1D**) and CD3+/CD57+ cells levels (22.4 ± 14.0% vs 15.4 ± 10.9%, p<0.0001, 22.4 ± 14.0% vs 14.4 ± 9.0%, p<0.0001 and 22.4 ± 14.0% vs 13.8 ± 9.3%, respectively, p<0.0001, **Figure 1E**), while no significant differences were found in Tγδ levels (**Figure 1F**).

### Treatment Related Effects on Immune System Cells in MM Patients

**Figure 2:**
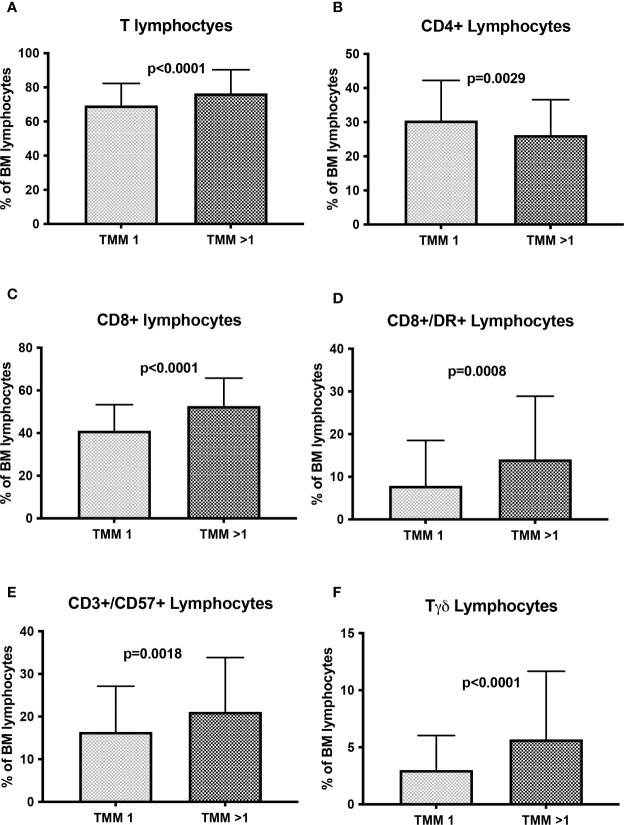
T cell subsets distribution in active MM patients according to treatment. The histograms show the distribution of T cell subsets in active MM patients according to the number of previous therapies (1 line or >1 lines). **(A)** total T cells. **(B)** CD4+ Lymphocytes. **(C)** CD8+ Lymphocytes. **(D)** CD8+/DR+ lymphocytes. **(E)** CD3+/CD57+ Lymphocytes. **(F)** Tγδ lymphocytes. The comparisons between mean percentages were made by T-test and results are expressed as mean ± standard deviation. TMM, treated MM.

Our results demonstrated that Myeloma treatment induces an immune cells distribution in MM patients. This was particularly evident in relationship to the number of previous lines of therapy. In fact, excluding samples of patients in whom bone marrow was performed within 3 months from ASCT (n=83), in patients who received >1 line of treatment a more pronounced cytotoxic T cell skewing was detected. In particular this latter subset of patients, compared to MM patients who received one line of therapy, displayed lower CD4+ lymphocytes (26.2 ± 10.4% vs 30.5 ± 10.7%, p=0.0029, **Figure 2B**) and higher total T cells (52.7 ± 13.1% vs 41.1 ± 12.2%, p<0.0001, **Figure 2A**), CD8+ lymphocytes (52.7 ± 13.1% vs 41.1 ± 12.2%, p<0.0001, **Figure 2C**), CD8+/DR+ lymphocytes (14.1 ± 14.8% vs 7.9 ± 10.6%, p=0.0008, **Figure 2D**), CD3+/CD57+ lymphocytes (21.1 ± 12.7% vs 16.4 ± 10.7%, p=0.0018, **Figure 2E**) and Tγδ cells (5.7 ± 6.0% vs 3.0 ± 3.0%, p<0.0001, **Figure 2F**).

### Impact of Novel Agents’ Treatment on Immune System

Therapy related information of treated patients are reported in **Table 2**. In our cohort most patients received a bortezomib based regimen as induction treatment, with lenalidomide generally restricted at patient relapse. To evaluate the impact of novel agents’ treatment on immune cells, we focused our attention on treated patients according to the specific backbone drug received during the induction therapy.

**Figure 3:**
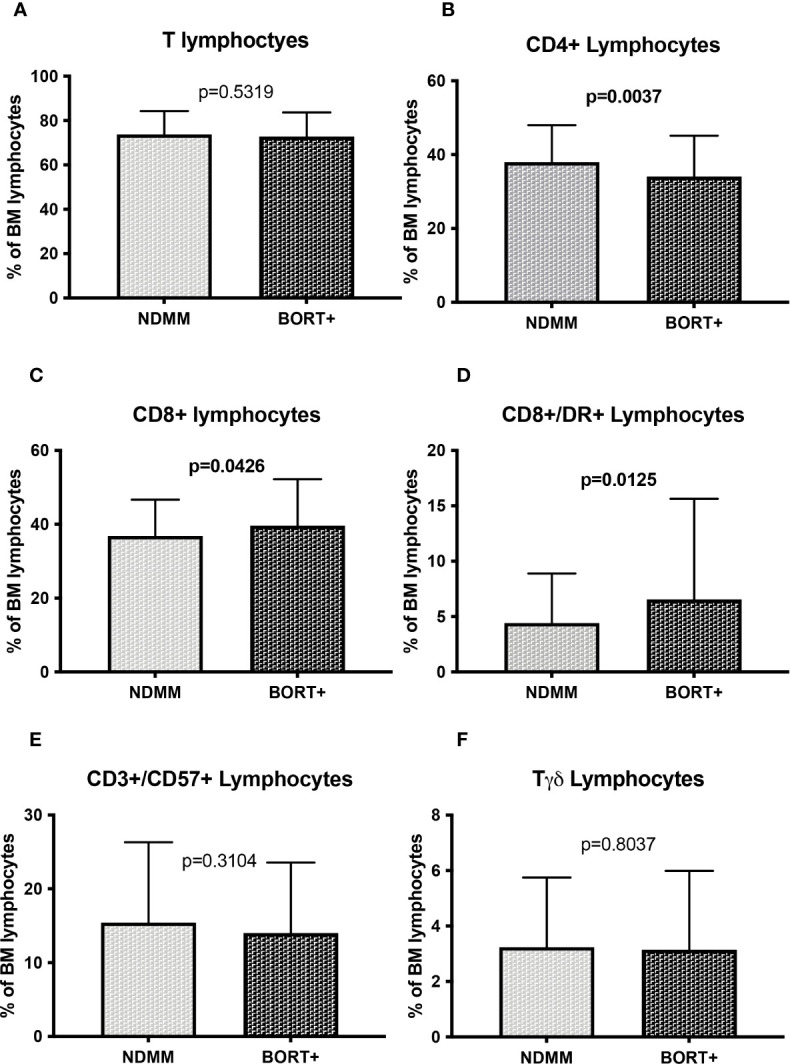
T cell subsets distribution in bortezomib treated MM patients **(A)** Total T cells; **(B)** CD4+ Lymphocytes; **(C)** CD8+ Lymphocytes; **(D)** CD8+/DR+ lymphocytes; **(E)** CD3+/CD57+ Lymphocytes; **(F)** Tγδ lymphocytes. The comparisons between mean percentages were made by T-test and results are expressed as mean ± standard deviation. NDMM, newly diagnosed MM; BORT, Bortezomib.

First of all, samples of NDMM patients were compared with a cohort of bortezomib based treated patients (n=77), in association with dexamethasone alone or to alkylating agents. To avoid misleading interpretations, patients who received ASCT and Immunomodulatory drugs (IMIDs) were excluded from the analysis. Although no significant differences in total T cells was found (72.8 ± 10.9% vs 73.7 ± 10.5%, p=0.5319, **Figure 3A**), bortezomib treatment led to a reduction of percentage of CD4+ cells (34.1% ± 11.1% vs 38.0 ± 9.9%, p=0.0037, **Figure 3B**) and to an increase of CD8+ (39.6 ± 12.6% vs 36.8 ± 9.9%, p=0.0426, **Figure 3C**), CD8+/DR+ (6.6 ± 9.1% vs 4.4 ± 4.5%, p=0.0125, **Figure 3D**) while no differences in CD3+/CD57+ lymphocytes (14.0 ± 9.6% vs 15.4 ± 10.9%, p=0.3104) and Tγδ lymphocytes were found (3.2 ± 2.9% vs 3.2 ± 2.5%, p=0.8037) (**Figures 3E, F**, respectively). In addition, in bortezomib treatment samples a reduction in total B lymphocytes (7.6 ± 6.2% vs 9.5 ± 6.4%, p=0.0182), CD19+/CD5- B cells (6.6 ± 5.8% vs 8.1 ± 5.4%, p=0.0522, **Supplementary Figures 2A, B**, respectively) and total NK cells (17.6 ± 11.8% vs 15.0 ± 8.4%, p=0.0283, **Supplementary Figure 2D**) was found while CD19+/CD5+ B cells and CD57+ NK cells were unaffected (1.5 ± 2.0 vs 1.5 ± 2.6, p=0.9286 and 9.1 ± 9.8% vs 7.7 ± 5.8%, p=01542, respectively, **Supplementary Figures 2C, E**, respectively).

**Figure 4:**
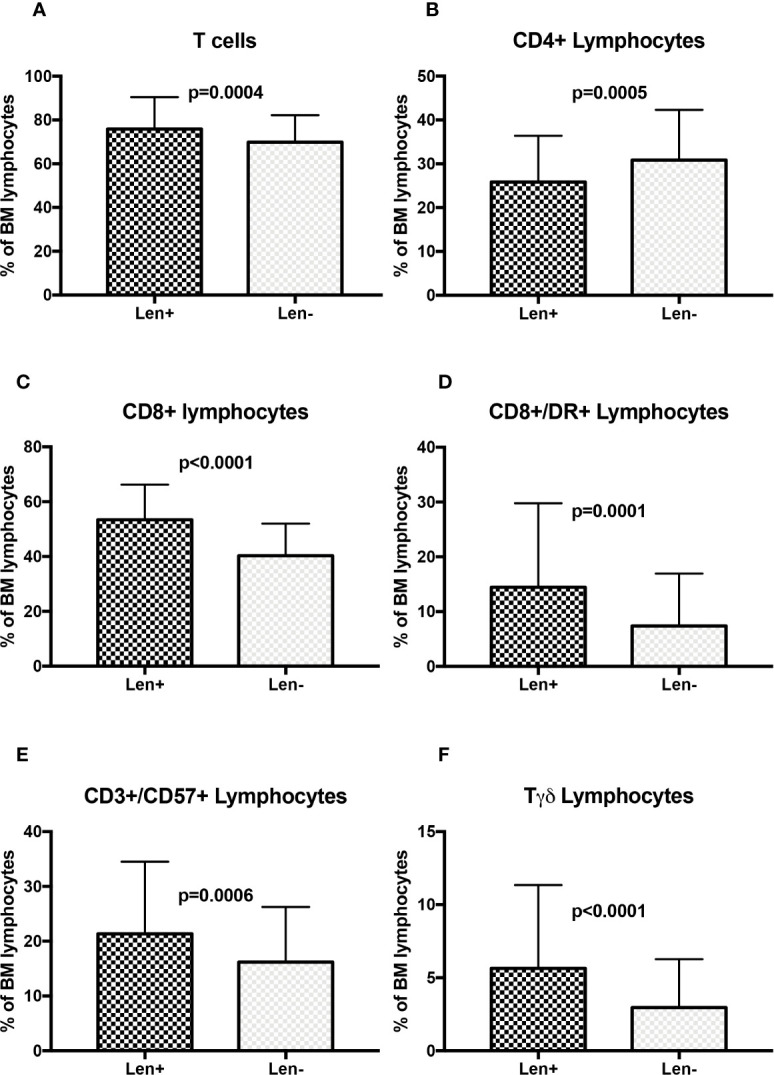
T cell subsets distribution in lenalidomide treated MM patients **(A)** Total T cells; **(B)** CD4+ Lymphocytes; **(C)** CD8+ Lymphocytes; **(D)** CD8+/DR+ lymphocytes; **(E)** CD3+/CD57+ Lymphocytes; **(F)** Tγδ lymphocytes. The comparisons between mean percentages were made by T-test and results are expressed as mean ± standard deviation. Len, Lenalidomide.

As far as lenalidomide treated patients samples (n=118) are concerned, a more pronounced cytotoxic skewing was found. In fact, lenalidomide towards untreated patients (n=130) was able to substantially increase total T cells (75.9 ± 14.6% vs 69.8 ± 12.4%, p=0.0004, **Figure 4 A**), CD8+ cells (53.5 ± 12.8% vs 40.3 ± 11.7%, p<0.0001, **Figure 4C**), CD8+/DR+ cells (14.4 ± 15.4% vs 7.4 ± 9.58%, p=0.0001, **Figure 4D**), CD3+/CD57+ cells (21.35 ± 13.2% vs 16.17 ± 10.1%, p=0.0006, **Figure 4E**) and Tγδ cells (5.6 ± 5.7% vs 3.0 ± 3.3%, p<0.0001, **Figure 4F**) and to reduce CD4+ cells (25.9 ± 10.5% vs 30.9 ± 11.4%, p=0.0005, **Figure 4B**), total B cells (5.8 ± 6.1% vs 11.3 ± 10.5%, p<0.0001), CD19+/CD5- B cells (6.0 ± 11.8% vs 9.3 ± 9.1%, p=0.0122) and CD19+/CD5+ cells (1.1 ± 2.3% vs 2.1 ± 2.9%, p=0.0063) (**Supplementary Figures 3A–C**). No significant effect on NK cells (15.9 ± 11.0% vs 16.6 ± 10.7%, p=0.6077) and CD57+ NK cells (7.5 ± 7.0% vs 8.2 ± 8.7%, p=0.4960) was documented (**Supplementary Figures 3D, E**, respectively).

### Effect of Autologous Stem Cell Transplantation on Immune Subsets Distribution

**Figure 5:**
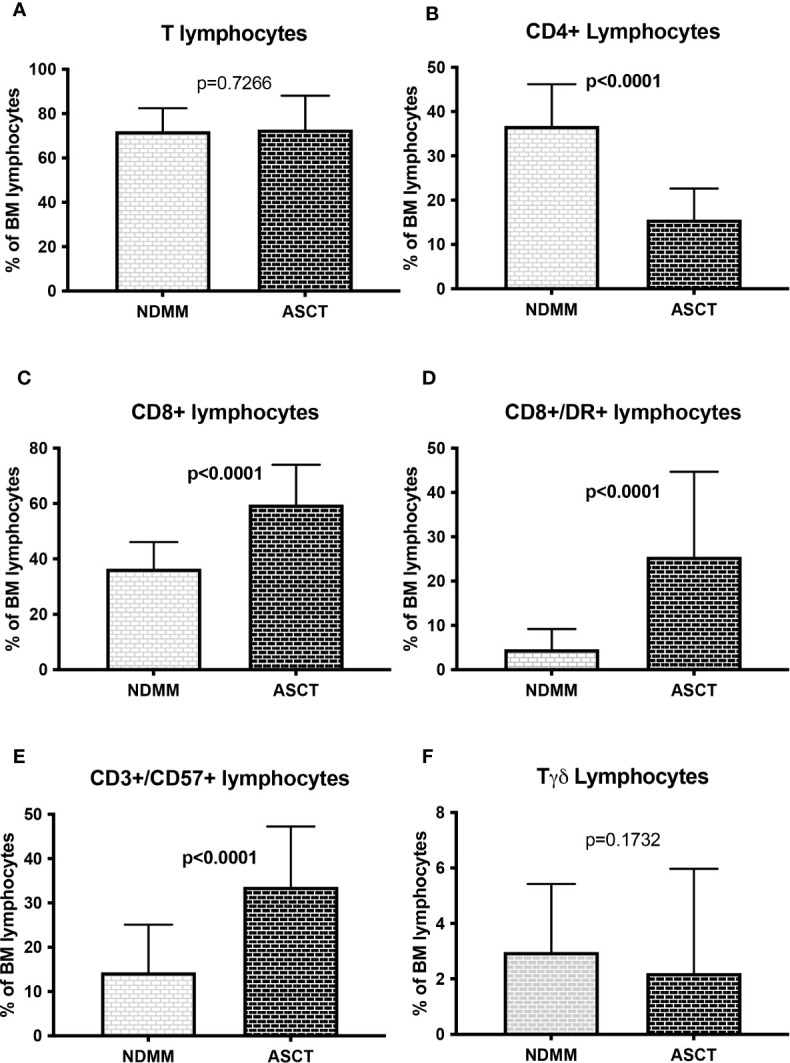
T cell subsets distribution in paired MM patients at diagnosis and after autologous stem cell transplantation (n=83) Samples of patients who received ASCT were collected within 3 months from conditioning regimen. **(A)** Total T cells. **(B)** CD4+ Lymphocytes. **(C)** CD8+ Lymphocytes. **(D)** CD8+/DR+ lymphocytes. **(E)** CD3+/CD57+ Lymphocytes. **(F)** Tγδ lymphocytes. The comparisons between mean percentages were made by T-test and results are expressed as mean ± standard deviation. NDMM, newly diagnosed MM; ASCT, autologous stem cell transplantation.

To evaluate the impact of ASCT on lymphocyte subsets distribution, 83/331 samples of treated patients were collected within 3 months from ASCT and compared with paired NDMM samples. All patients received a bortezomib based induction regiment, either in association with alkylating agents or thalidomide. Even though no differences were found in total T cells levels (72.1 ± 10.3% vs 72.8 ± 15.3%, p=0.7266, **Figure 5A**), a cytotoxic T- cell skewing was confirmed in patients who received ASCT, with reduction of CD4+ cells percentage (15.6 ± 7.0% vs 36.8 ± 9.4.%, p<0.0001, **Figure 5B**) and increase of CD8+ lymphocytes (59.7 ± 14.4% vs 36.5 ± 9.6%, p<0.0001, **Figure 5C**), CD8/DR+ lymphocytes (25.5 ± 19.2% vs 4.6 ± 4.6%, p<0.0001, **Figure 5D**) and CD3+/CD57+ lymphocytes levels (33.7 ± 13.6% vs 14.3 ± 10.8%, p<0.0001, **Figure 5E**) while no differences in Tγδ cells was evidenced (2.2 ± 3.7% vs 3.0 ± 2.5%, p=0.1732, **Figure 5F**). At variance, a significant reduction of NK cells (11.6 ± 7.6% vs 14.7 ± 6.8%, p=0.0135) and CD57+ NK cells (4.3 ± 4.0% vs 6.9 ± 4.9%, p=0.0003) was found (**Supplementary Figures 4A, B**, respectively). Finally, a B cell subsets modulation with reduction of CD19+/CD5- B cells (6.5 ± 7.2% vs 9.5 ± 6.1%, p=0.0037) and increase of CD19+/CD5+ B cells (6.3 ± 5.5% vs 2.1 ± 3.7%, p<0.0001 was evident in patients who received ASCT (**Supplementary Figures 4C–E**).

**Figure 6:**
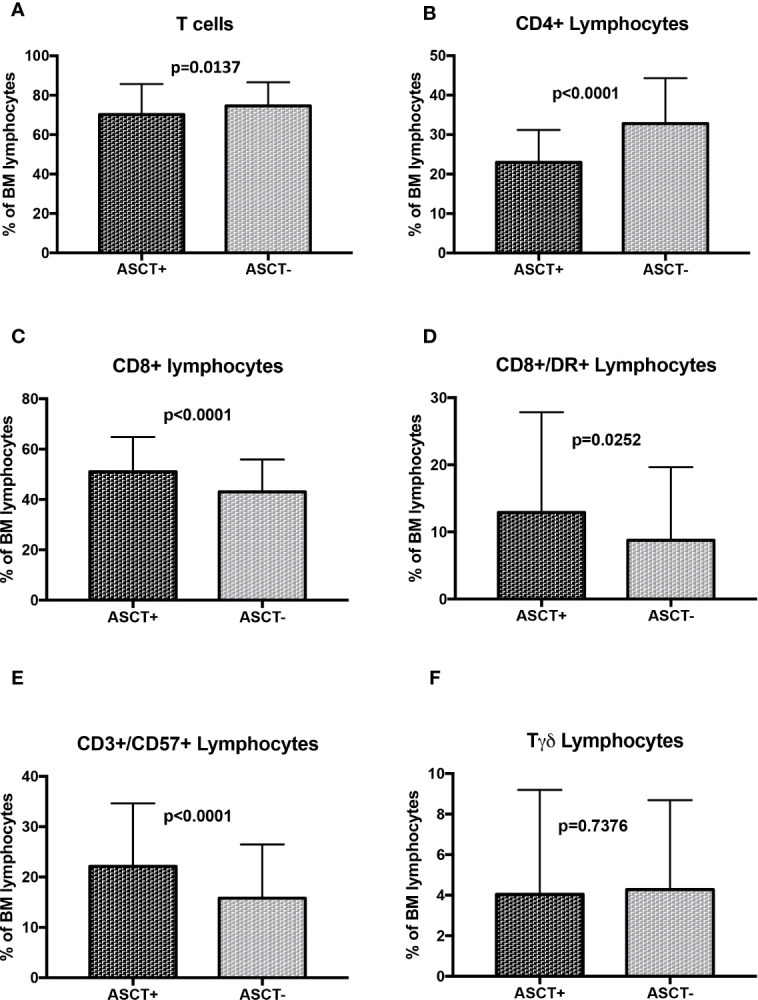
T cell subsets distribution in MM treated patients with autologous stem cell transplantation (n=110) as compared to patients treated without autologous stem cell transplantation (n=138). **(A)** Total T cells; **(B)** CD4+ Lymphocytes; **(C)** CD8+ Lymphocytes; **(D)** CD8+/DR+ lymphocytes; **(E)** CD3+/CD57+ Lymphocytes; **(F)** Tγδ lymphocytes. The comparisons between mean percentages were made by T-test and results are expressed as mean ± standard deviation. ASCT, autologous stem cell transplantation.

To avoid possible misleading effects due to viral triggers (i.e. CMV and/or EBV reactivation) or immune reconstitution after ASCT, we evaluated the same immune subsets in treated patients excluding the 83 samples collected close to ASCT. In the samples of these patients who received ASCT (n=110) with respect to patients who did not (n=138) we observed a significant reduction in total T cells (70.3 ± 15.4% vs 74.6 ± 12.0%, p=0.0137, **Figure 6A**) and CD4+ cells (23.0 ± 8.2% vs 32.8 ± 11.5%, p<0.0001, **Figure 6B**) and confirmed a cytotoxic T cell skewing with increase in CD8+ cells (51.0 ± 13.8% vs 43.0 ± 12.9%, p<0.0001, **Figure 6C**), CD8+/DR+ cells (12.9 ± 14.9% vs 8.8 ± 10.9%, p=0.0252, **Figure 6D**) and CD3+/CD57+ cells (22.2 ± 12.5% vs 15.8 ± 10.7%, p<0.0001, **Figure 6E**), while no significant differences in Tγδ levels (4.1 ± 5.2% vs 4.3 ± 4.4%, p=0.7376)(**Figure 6F**) NK cells (15.6 ± 11.1% vs 16.6 ± 10.6%, p=0.5752) and CD57+ NK cells were found (7.5 ± 7.2% vs 8.2 ± 8.5%, p=0.5152) (**Supplementary Figures 5A, B**). As reported before, also B cells were higher in ASCT+ patients (11.43 ± 11.0% vs 6.5 ± 6.6%, p<0.0001), with significant increase in CD19+/CD5- B cells (4.6± 4.8% vs 2.1± 2.8%, p<0.0001) and an almost significant increase of CD19+/CD5+ B cells (1.9 ± 3.1% vs 1.3 ± 2.3%, p=0.0589) (**Supplementary Figures 5C–E**).

## Discussion

In our large series of cases, we demonstrated a relevant therapy related T cell modulation in active Multiple Myeloma patients, with a cytotoxic T cell polarization resulting from Myeloma treatment. More specifically, in treated patients a reduction of CD4+ T cells and an increase in CD8+, CD8+/DR+ and CD3+/CD57+ T cell was observed with respect to newly diagnosed patients, these changes being more pronounced when referred to the number of previous therapies.

The possibility to collect bone marrow data from this large series of patients undoubtedly is the strength of our study but at the same time, due to the impossibility to perform more accurate evaluations in such a broad period of observations, its numerosity represents a weakness of our piece. These shortcomings include the lack of functional studies and the extensive characterization of CD4+ and CD8+ T lymphocytes subsets and their properties, the different time points of samples collections and the heterogeneity of treatment received. However, to our knowledge, an immune subset characterization of a large cohort of patients affected by plasma cell dyscrasias is still lacking, even more outside clinical trials. Therefore, our study represents a reliable real-life picture of the immune system distribution in patients affected by plasma cell dyscrasias.

Considering the different treatments responsible for these variations, ASCT represents one of the main candidates. Up to now most efficacy of ASCT in MM is likely to be the consequence of high dose chemotherapy, but several hints suggest something more than a mere cytoreductive effect. In fact, accumulating evidence indicates that the melphalan used in conditioning regimen induces a pro-inflammatory cytokine burst and disrupts the immune-suppressive tumor micro-environment (23–25). Our results reporting a switch of T cells toward a cytotoxic phenotype are consistent with a distinctive immunological activity of ASCT. These effects are not transiently induced by immune reconstitution and/or a viral stimulation, since the same changes can be observed also far from the time of ASCT, thus implying a more profound and persistent effect of transplantation in immune subsets distribution. Most importantly, a similar cytotoxic polarization with reduction of CD4+ cells and increase in CD8+ effector memory cells (resembling the CD3+/CD57+ cells analyzed in our cohort) was observed in patients with long term complete response after ASCT, suggesting a putative immune surveillance effect (18). Of notice, since NK cells are reduced immediately after ASCT, strategies including elotuzumab, whose mechanism of action is fully dependent on NK cells activity, as consolidation after transplantation might be not effective (26). Otherwise, adoptive cell therapy strategies based on NK cells can represent an interesting choice in MM treatment. In fact, chimeric antigen receptor NK cells (CAR-NK) therapy owns several advantages towards CAR-T cells counterpart, more specifically an off-the shelf availability with absence of graft versus host disease, lower risk of cytokine release syndrome and multiple mechanism of tumor recognition (27). Preclinical data of NKG2D and BCMA CAR-NK cells showed efficacy to eradicate MM cells and clinical trials using CD19 CAR-NK and BCMA CAR-NK are now enrolling (28).

Novel agents like proteasome inhibitors and IMIDs completely changed the landscape of Multiple Myeloma treatment improving patient’s survival. Most of them acts directly on myelomatous plasma cells but, especially for IMIDs, an indirect activity through immune system has been postulated (29–32). In our cohort we evaluated the consequence of bortezomib and lenalidomide treatment on immune subsets distribution. As expected, lenalidomide treatment was able to induce a marked cytotoxic T cell skewing without affecting NK cells, besides these effects were also shown following bortezomib treatment, even if less pronounced. Up to now, lenalidomide represents the backbone of the most effective treatments used in patients with newly diagnosed (33–35) and relapsed Multiple Myeloma (36–39); moreover, lenalidomide maintenance after ASCT is the standard of care for young patients. Considering the remarkable cytotoxic polarization induced by these treatments, our results offer a further strong rationale for lenalidomide maintenance, in particular after ASCT (40).

Tγδ cells are a small subset of lymphocytes recognizing small non-peptidic phosphorylated antigens, moreover, the activation of Vγ9Tγδ cells by amino bisphosphonates has been demonstrated (41). In our cohort, a significant increase of Tγδ cells was not observed, except for lenalidomide treated patients. However, almost all patients received bisphosphonates for the treatment of Myeloma bone disease, thus entirely attributing this increase to lenalidomide treatment itself.

Immuneparesis is an established progression risk factor in MGUS patients (11) and patients with active MM are often characterized by hypogammaglobulinemia (42). In our cohort, only treated MM patients showed significative B cell reduction, including following bortezomib based and lenalidomide based treatments and ASCT. Consequently, at least in the first step of MM pathogenesis, most B cell dysfunction is qualitative rather than quantitative. In addition, no significant quantitative differences were found also in total NK cells, confirming that, in MM progression process, NK cells are rather dysfunctional than reduced. Several lines of evidence support this hypothesis, including an altered balance in favor of immunosuppressive cytokine like Interleukin 10 and TGF-β (43, 44), NK suppression *via* NKG2D down-regulation by soluble MICA and MICB (45) and by upregulation of HLA-1 on pathological plasma cells (46) and acquisition of “exhausted” phenotype signature with up-regulation of programmed death receptor-1 (PD-1) (47).

Given the complexity of tumor microenvironment and the numerous subsets of bone marrow cells involved in MM biology, novel multiparametric cytometry approaches like mass cytometry by time-of flight (CytOF) were recently introduced. Using this approach, Kourelis et al. identified 12 immune clusters within the CD45+ compartment of patients with PCD. These authors also demonstrated that the tumor microenvironment of light chain amyloidosis is completely different as compared to other PCD and that treatments, including ASCT, promote the activation of several immune suppressive populations (48). Furthermore, they recently suggested that tumor immune microenvironment features could be used to guide rational selection of post-ASCT maintenance/consolidation strategies in MM patients (49).

With the advent of novel immunotherapeutic approaches likes antibody drug conjugated, bispecific antibodies (50), chimeric antigen receptor T and NK cells (51, 52), a comprehensive analysis of immune system is mandatory to assess treatment efficacy and drug synergy, therefore novel high throughput analysis as CytOF or single cell RNA sequencing are helpful to rule out these issues. Given the retrospective nature and the broad time period of our study, in this study we reported only a limited immune subsets descriptive analysis, nevertheless the large study cohort and the real-life evaluation represent somehow a novelty in the field of Multiple Myeloma immunology.

In conclusion, we demonstrated that AMM patients are characterized by a profound T cell modulation as compared to MGUS and SMM patients and that most of these changes are therapy related. Among the different therapeutic strategies commonly used in MM, ASCT and lenalidomide treatments polarize immune system toward a dominant cytotoxic response, likely contributing to the anti-Myeloma effect of these regimens.

## Data Availability Statement

The raw data supporting the conclusions of this article will be made available by the authors, without undue reservation.

## Ethics Statement

The studies involving human participants were reviewed and approved by Institutional Review Board of Azienda Ospedaliera di Padova. The patients/participants provided their written informed consent to participate in this study.

## Author Contributions 

GB designed the research, analyzed data and wrote the manuscript. LP, SV, TB and MS provided patient’s samples and patient’s data. VT and SC performed flow analysis. AT, GC, CV, VG and AF contributed to collect and prepare samples for flow analysis. MF, SM and FP contributed to analyze data. GS provided funding, participated to the analysis of data and critically reviewed and edited the manuscript. RZ designed the study, analyzed data, wrote the manuscript and supervised the study. All authors contributed to the article and approved the submitted version.

## Conflict of Interest

The authors declare that the research was conducted in the absence of any commercial or financial relationships that could be construed as a potential conflict of interest.
