# Supplementary material for: Treatment Induced Cytotoxic T-Cell Modulation in Multiple Myeloma Patients
Source: Front Oncol. 2021 Jun 15;11:682658. doi: 10.3389/fonc.2021.682658 (PMC8239308; doi:10.3389/fonc.2021.682658)

SUPPLEMENTARY

Figure 1: B and NK cells distribution in MGUS, sMM, nMM and tMM samples.
Panel A: total B cells. **Panel B:** CD19+/CD5- B cells. **Panel C** CD19+/CD5+ B cells. **Panel D:** total NK cells. **Panel E:** CD57+ NK cells.
The comparisons between mean percentages were made by Anova followed by Tukey's multiple comparison test. For all plots data are expressed as mean ± standard deviation.
Abbreviations. MGUS: monoclonal gammopathy of undetermined significance. sMM: smouldering Multiple Myeloma. nMM: newly diagnosed Multiple Myeloma. tMM: treated Multiple Myeloma.

Figure 2: B and NK cells distribution in bortezomib treated patients.
Panel A: total B cells. **Panel B:** CD19+/CD5- B cells. **Panel C:** CD19+/CD5+ B cells. **Panel D:** total NK cells. **Panel E:** CD57+ NK cells.
The comparisons between mean percentages were made by T-test and results are expressed as mean ± standard deviation.
Abbreviations. nMM: newly diagnosed MM. Bort: Bortezomib.

Figure 3: B and NK cells distribution in lenalidomide treated patients.
Panel A: total B cells. **Panel B:** CD19+/CD5- B cells. **Panel C:** CD19+/CD5+ B cells. **Panel D:** total NK cells. **Panel E:** CD57+ NK cells.
The comparisons between mean percentages were made by T-test and results are expressed as mean ± standard deviation.
Abbreviation. Len: Lenalidomide.

Figure 4: B and NK cells distribution in patients receiving autologous stem cell transplantation
Panel A: total NK cells. **Panel B:** CD57+ NK cells. **Panel C**: total B cells. **Panel D:** CD19+/CD5- B cells. **Panel E:** CD19+/CD5+ B cells.
The comparisons between mean percentages were made by T-test and results are expressed as mean ± standard deviation.
Abbreviations. nMM: newly diagnosed MM. ASCT: autologous stem cell transplantation.

Figure 5: B and NK cells distribution in patients treated with autologous stem cell transplantation
Panel A: total NK cells. **Panel B:** CD57+ NK cells. **Panel C**: total B cells. **Panel D:** CD19+/CD5- B cells. **Panel E:** CD19+/CD5+ B cells.
The comparisons between mean percentages were made by T-test and results are expressed as mean ± standard deviation.
Abbreviation. ASCT: autologous stem cell transplantation

Supplementary Figure 1

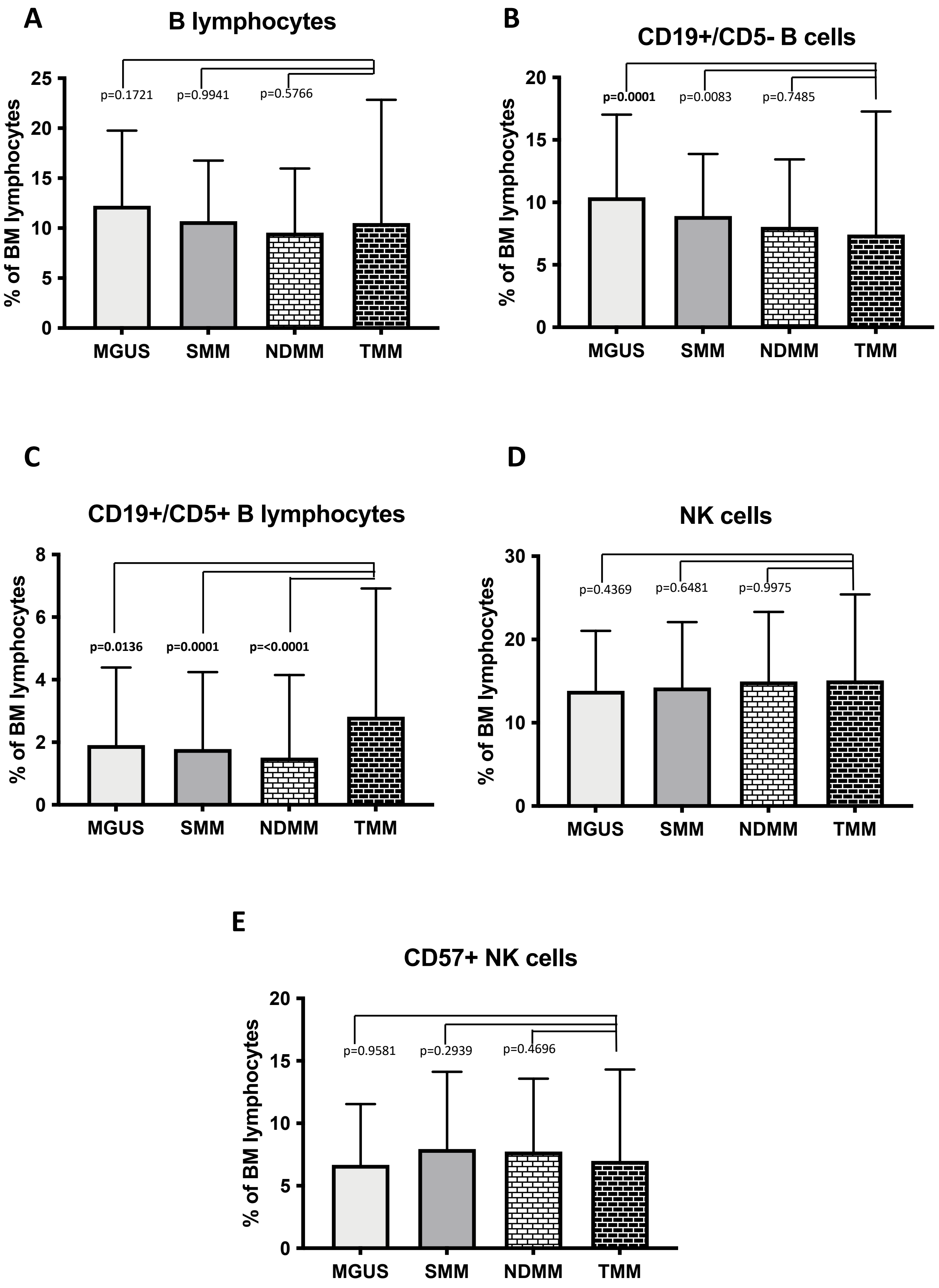

Supplementary Figure 2

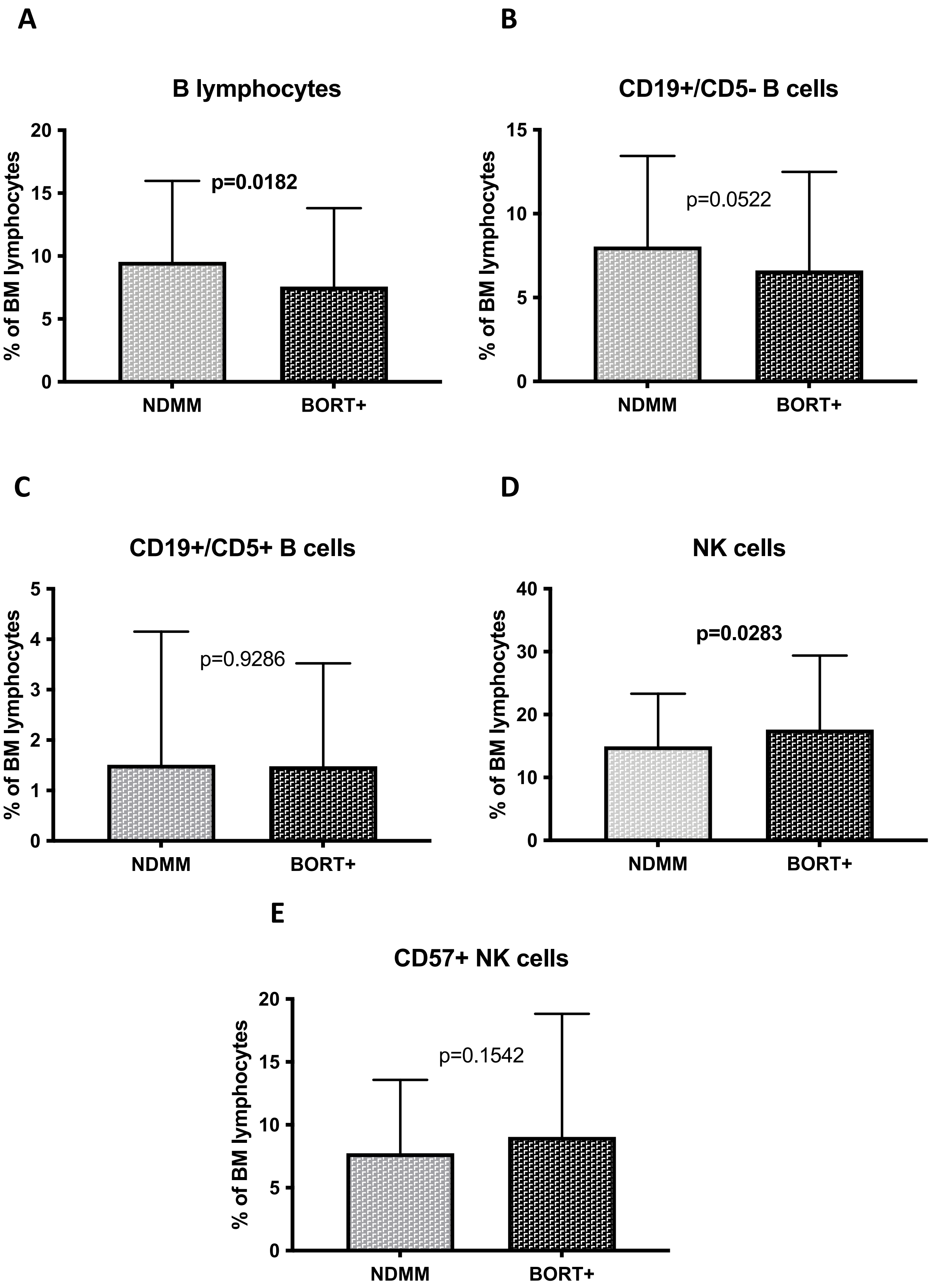

Supplementary Figure 3

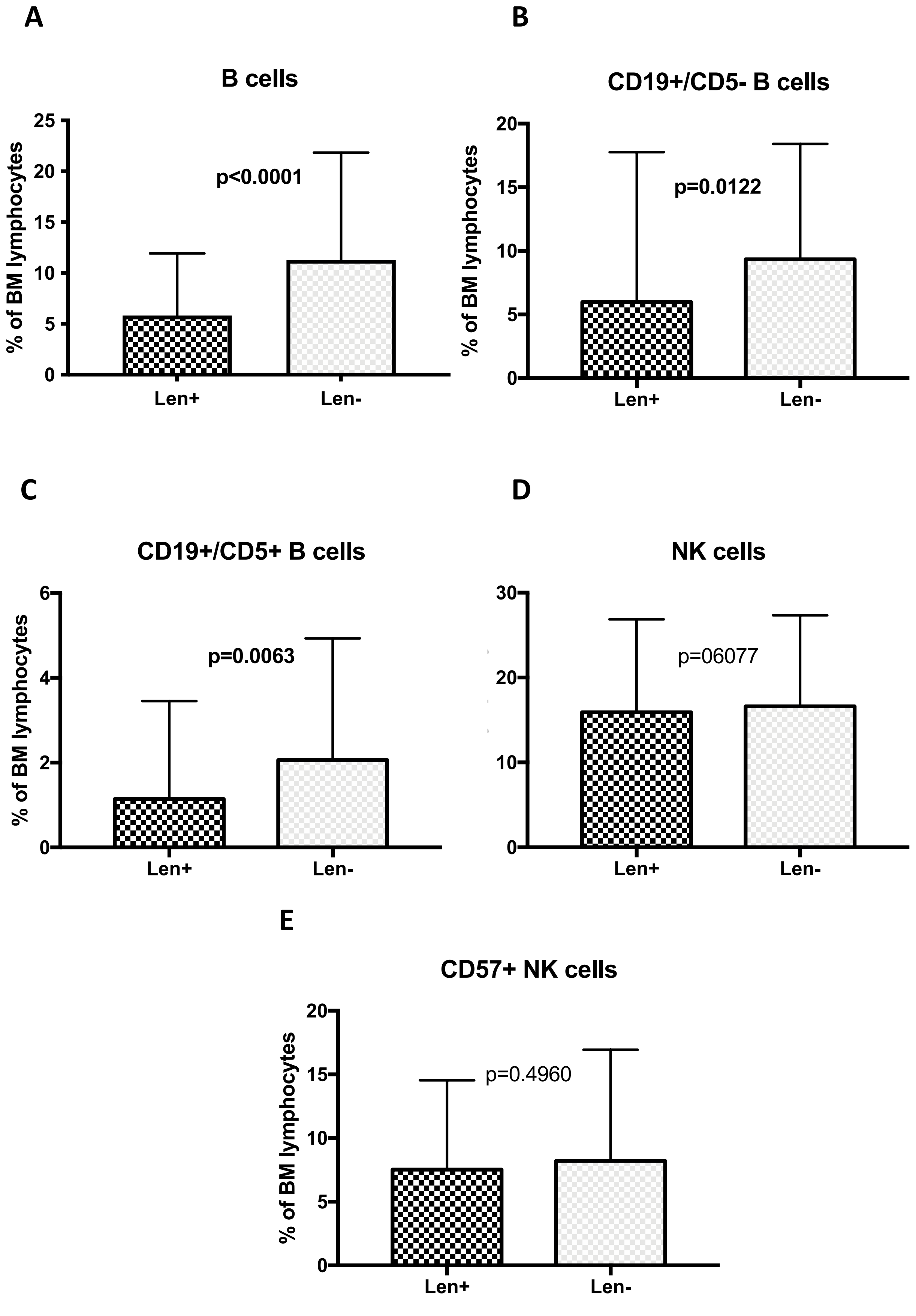

Supplementary Figure 4

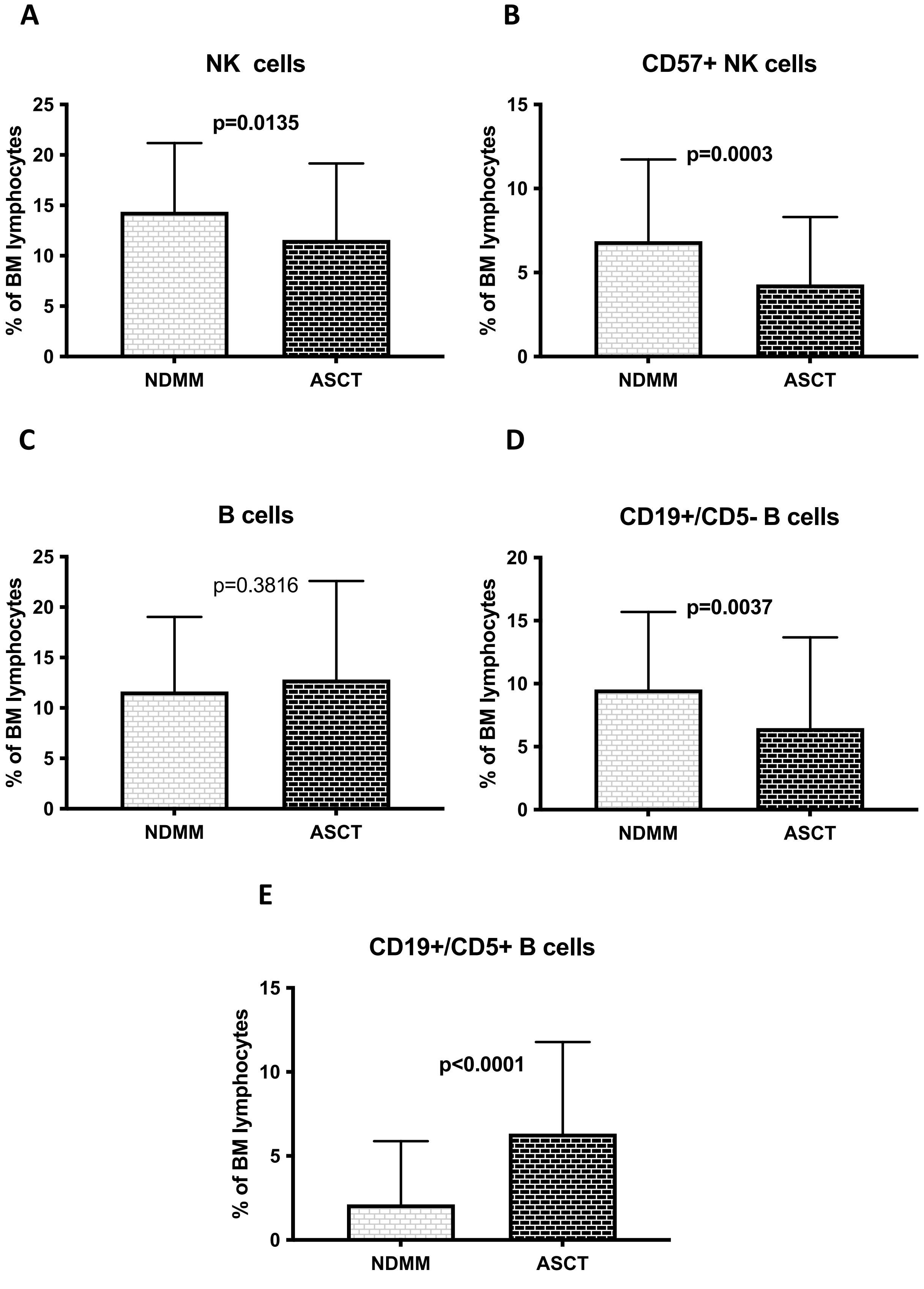

Supplementary Figure 5

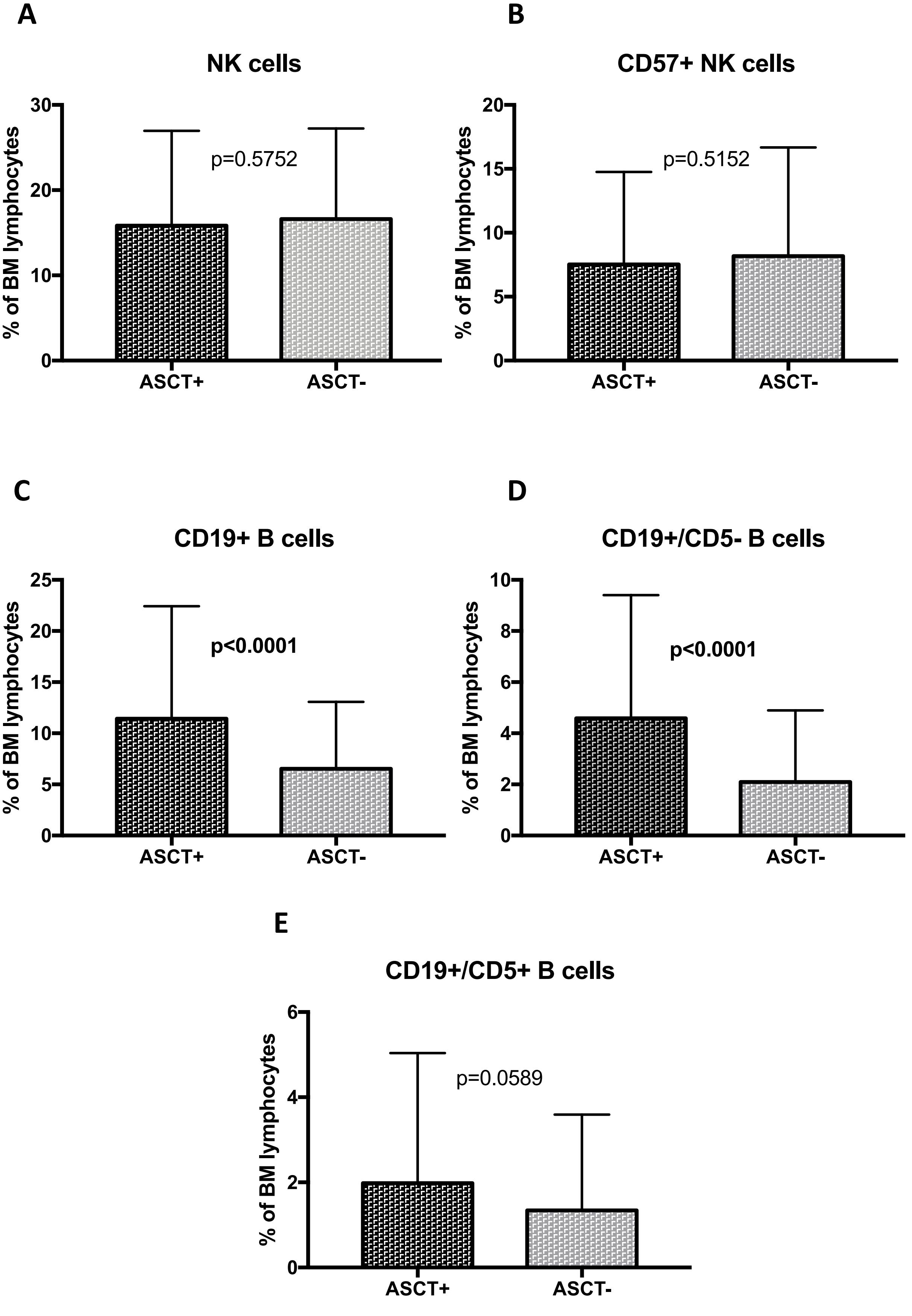

Supplement: Supplementary file 1 [file DataSheet_1.pdf]
